# *Toxoplasma gondii* survey in cats from two environments of the city of Rio de Janeiro, Brazil by Modified Agglutination Test on sera and filter-paper

**DOI:** 10.1186/s13071-017-2017-8

**Published:** 2017-02-17

**Authors:** Paula F. Bolais, Philippe Vignoles, Pamela F. Pereira, Rafael Keim, Abdelkrim Aroussi, Khadja Ismail, Marie-Laure Dardé, Maria Regina Amendoeira, Aurélien Mercier

**Affiliations:** 1 Univ. Limoges, INSERM UMR-S 1094 Tropical Neuroepidemiology, Institute of Neuroepidemiology and Tropical Neurology, 2 Rue du Dr. Marcland, Limoges, 87025 France; 2 Toxoplasmosis and other Protozoosis Laboratory of Oswaldo Cruz Institute, Av. Brasil 4365, Rio de Janeiro, 21045-900 Brazil; 3 Quatro Elementos Veterinary Medicine and Environmental consulting, Rua Coronel Moreira Cesar, 211 Bl. 2/502, Niteroi, 24.230-052 Brazil

**Keywords:** *Toxoplasma gondii*, Cats, Serology, Modified agglutination test, Filter paper, Eco-epidemiology, Brazil

## Abstract

**Background:**

*Toxoplasma gondii* is a protozoan with a worldwide distribution, in warm-blood animals, including humans. Local conditions and environmental disturbances may influence transmission dynamics of a zoonotic agent. This study evaluates the epidemiology of *T. gondii* based on toxoplasmosis prevalence in two populations of cats living in distinct urbanization conditions in Rio de Janeiro, Brazil.

**Methods:**

Among 372 domestic cats sampled, 265 were from a public shelter located downtown Rio and 107 from a relatively preserved wild environment in a residential area. Sera and eluates from dried blood spots were tested for detection of IgG antibodies against *T. gondii* by modified agglutination test (MAT).

**Results:**

Antibodies to *T. gondii* were detected in 32/265 (12.08%) animals from the public shelter and in 4/107 (3.74%) cats from the residential area. Identical results were observed for sera and eluates.

**Conclusions:**

Filter paper provides a reliable accurate alternative storage option when conditions of sample collection and transportation in the field are unfavorable. The significantly lower prevalence in the residential area is discussed in terms of environmental, biological and behavioral features.

## Background

*Toxoplasma gondii* is a protozoan with a successful worldwide distribution and a widespread presence in warm-blood animals, including humans [1]. These characteristics have encouraged numerous studies in order to better understand its biology, genetics and transmission dynamics. The life-cycle of *Toxoplasma gondii* comprises three infective stages: the invasive tachyzoites, the encysted bradyzoites and the environmental sporozoites protected by the oocyst wall. Acting as definitive hosts, either domestic cats or wild felids play the major role in spreading the parasite by shedding oocysts in faeces [2]. Contaminated soil, water courses and agricultural crops become the source of infection for domestic or wild animals and livestock. For humans, the most important routes of transmission are through ingestion of undercooked meat with cysts, poorly washed vegetables and water or soil contaminated with oocysts [3, 4].

Globally, the prevalence of human *Toxoplasma* infection varies significantly. It ranges from less than 10% in Korea [5] to over 80% in Brazil [6]. These differences are attributed to risk factors that may vary between regions, such as the type of food, the cooking mode, adequate water treatment and intensity of environmental exposure [4, 6–10]. Although infection with *T. gondii* is generally asymptomatic, human infection in South America, mainly in Brazil, may lead to neurological complications and ocular lesions, probably due to the genetic difference in these virulent strains and poor host adaptation [11]. With significant serological prevalence of *T. gondii* in the Brazilian general population (up to 80%) [6], the national government faces a large public health burden in view of many cases of congenital toxoplasmosis: 6 to 9/10,000 births in Brazil [12, 13] compared to 3.3/10,000 births in France [14].

In veterinary medicine worldwide, *T. gondii* is a major cause of abortion in sheep and goats [15]. Clinical toxoplasmosis and fatal cases of the disease have been reported in New World monkeys [16], squirrels, New World porcupines [17], pigs [18], birds [19–22] and marine mammals [23–25]. Taking into account only recent studies using the same serological test (modified agglutination test) in animal samples from the Brazilian environment, it is possible to observe that, regardless of their habitats, several animal species have a high prevalence of *T. gondii* antibodies: up to 87% (55/63) of domestic cats [26] (Table 1), 86% (99/115) of pigs [27], 53.3% (202/379) of sheep [28], 75% (48/64) of capybaras [29], 60% (10/18; 60/37) of pacas [30, 31], 85.3% (99/116) of non-human primates [32], 79.7% (157/197) of cattle egrets [33] and 86.3% (82/96) of Amazon river dolphins [34]. These data, in addition to those concerning the high prevalence in the Brazilian human population, suggest that the country has a high environmental contamination.

**Table 1:** Studies conducted in Brazil on *Toxoplasma* seroprevalence in cat populations using the MAT technique

Location^a^	% positive (No. positive/total no.)	Cut-off titer	Cat life style	Reference
Pernambuco	66.6 (32/48)	1:25	free-roaming	[33]
Pernambuco	44.4 (20/45)	1:25	owned	[33]
Rondônia	87.3 (55/63)	1:25	free-roaming	[26]
São Paulo	35.4 (84/237)	1:25	free-roaming	[74]
Paraná	84.4 (49/58)	1:20	owned	[75]
São Paulo	19 (19/100)	1:16	NI	[76]
São Paulo	26.3 (132/502)	1:20	free-roaming	[76]

*NI* not informed

^a^State of Brazil

Previous studies have demonstrated that local conditions and environmental disturbances may influence the genetic composition of a zoonotic agent [35] or its transmission dynamics [36]. Our study aimed to verify if environmental differences were likely to influence *T. gondii* epidemiology in two populations of cats living in quite distinct conditions in the city of Rio de Janeiro. This will help us to better understand the causal relationships between urban areas, biological diversity and *T. gondii* prevalence and then provide useful information for decision-making in public health.

Furthermore, we verified the accuracy of the serological testing with the use of samples stored on filter papers, in addition to the classic MAT method on serum. Collection cards have been used for epidemiological surveys in wildlife [37, 38], and more specifically for screening of *T. gondii* antibodies in peccaries, brocket deer and lowland tapir [39], as well as in wild goose [40], red foxes [41], wild waterfowl [42], beavers [43], and commensal rodents [44]. Indeed, this is the first time that dried blood spots of cat samples have evaluated as suitable to MAT.

## Methods

### Study area

**Fig. 1:**
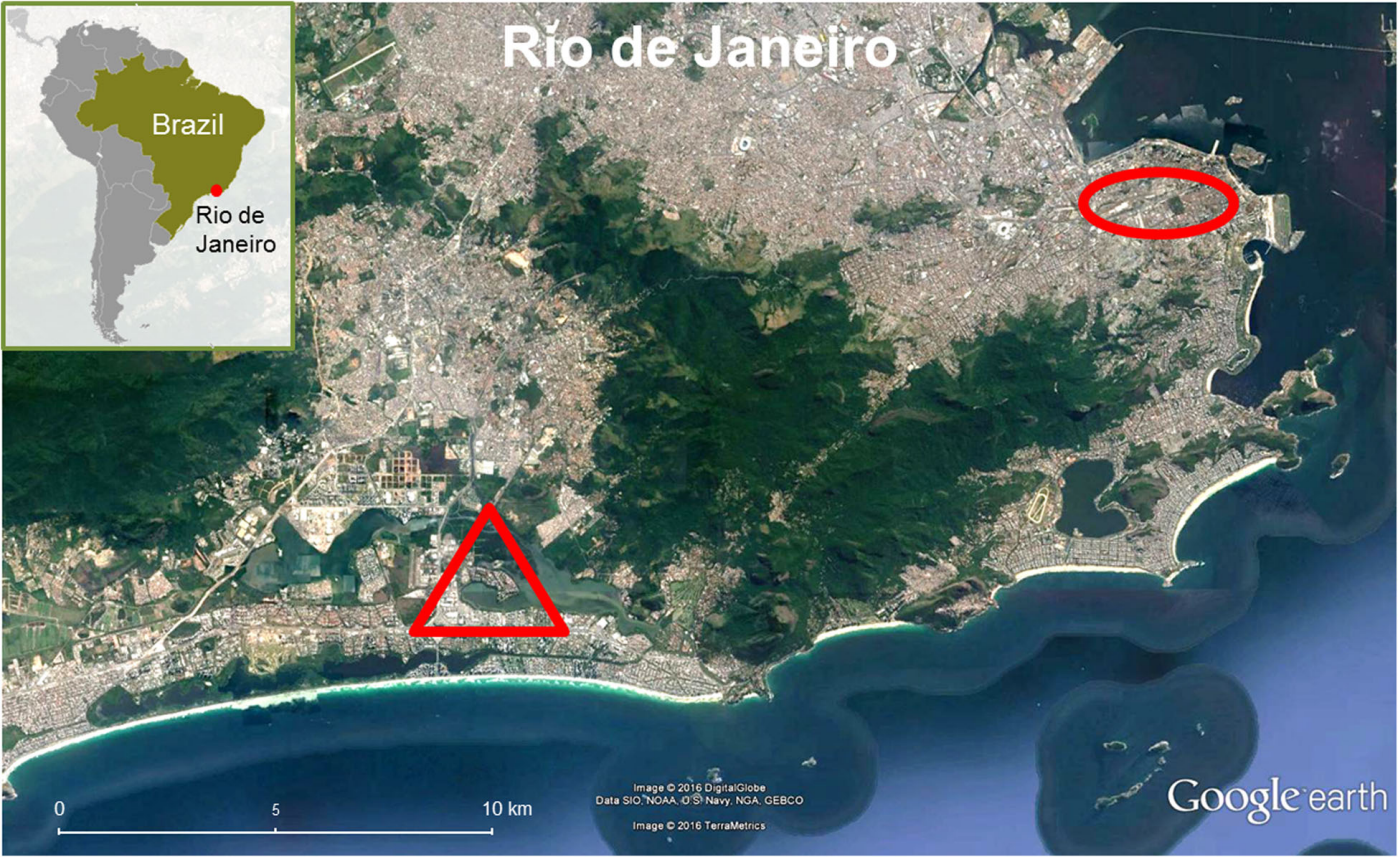
The city of Rio de Janeiro and sample collection areas. The municipal shelter is located within the *red circular* area, and the residential district corresponds to the *red triangular* area. Map: Adapted from the Central Intelligence Agency Web site [77] and GOOGLE EARTH - Data SIO, NOAA, U.S. Navy, NGA, GEBCO, image© 2016 DigitalGlobe/TerraMetrics

The municipality of Rio de Janeiro is located in a humid tropical marine-plain climate in southeast Brazil. It has an area of about 122,000 hectares and a population of over 6 million people [45]. The demographic density in city districts varies significantly. Taking this into account, two very different places in the city of Rio de Janeiro were chosen for sampling: a public cat shelter located downtown and a private residential area situated in a seaside district (Fig. 1). Both sites are currently classified as urban areas. Despite this classification, in the shelter area urbanization is older and more intense than in the residential district.

The municipal animal shelter (MS) is located downtown. It has an area of 9300 m^2^, harbors 300 cats, and provides full-time veterinary monitoring and commercial cat food. Colonization of the country began in the seventeenth century in this part of the city. Urbanization occurred gradually until the mid-nineteenth century when settlements grew increasingly faster. This triggered significant urbanization of the city resulting in drainage, embankment and landfill of its original swamps and marshes, and transformed its landscape to its current condition (99% of its territory is classified as urbanized) with a demographic density of 100 people per hectare [46] (Table 2).

**Table 2:** Environmental characteristics of sample collection areas and living conditions of the corresponding cat populations

Features^a^	Sample collection areas	
	Municipal shelter	Residential district
Living space	shelter	private residential district
Life style	sheltered	free-roaming (stray and feral)
Food habits	mostly kibbles	mostly prey
Area (m^2^)	9,300	780,000
Cat density (m^2^ per cat)	31	3,587
Biodiversity	poor	rich
Climate	humid tropical	humid subtropical
Annual rainfall index (mm)^b^	512.2	966.2
Landscape	embankment, landfill	sandbank, mangroves
District urbanization (%)	99	55
District density (people per hectare)	up to 100	up to 50

^a^Cat population data were provided by the Special Office of Defense and Promotion of Animals of the City of Rio de Janeiro and by Quatro Elementos Veterinary Medicine and Environmental consulting. The climatic and demographic data are available at http://www.armazemdedados.rio.rj.gov.br/

^b^Data from the 2014 report of Alerta-Rio [69]

The residential seaside district is less than 20 km from downtown, and has a demographic density of less than 50 people per hectare [47]. It is composed of barrier islands (sandy strips) separated from the mainland by a wide lagoon complex. Until recently, this was a well-preserved region, being submitted to a systematic urbanization from the 1970s. Currently, only 55% of its area is classified as urbanized. The private residential district (RD), where the population of free-range cats sampled for this study lives, is located in a peninsular region. It has an area of 780,000 m^2^, of which 18% are buildings whose construction took place in the 2000s. They are surrounded by a lagoon with high salinity and 1.4 m depth maximum, and water temperatures range from 17 to 34 °C according to the season of the year. The lagoon preserves in its surroundings a wide area of mangroves, and is contiguous with areas of preserved sandbanks and flooded fields. The mangroves are home to high biodiversity, with fish stocks, crustaceans and mollusks. These provide an abundant food supply which attracts numerous birds and mammals (8 species of wild mammals, 32 of birds and 4 of reptiles) (Rafael Keim, pers. comm.). This wild fauna shares the territory with about 14,000 human inhabitants and their pets and an estimated population of 200 stray cats. This estimation, made by a local veterinarian, was obtained by counting all the cats in random samples of district sub-regions and extrapolating the count to the whole district.

### Sample collection

In 2014 and 2015, a total of 372 cats from these two different populations of Rio de Janeiro were sampled for this study: 265 from the public cat shelter located in city center which gathered cats from a surrounding public square and 107 from the private residential area situated in the seaside district. Each animal from both populations had been previously identified with subcutaneous electronic microchips (AnimallTAG®) and had individual information recorded (sex, morphologically estimated age, reproductive status). Their blood was collected by puncture of the cephalic vein and four to six drops of the collected fluid were used to soak two circles of a *Whatman™903* specimen collection paper (Whatman*™* GE Healthcare Life Sciences, Florham Park, USA). Remaining collected blood was stored in collection tubes without anticoagulant to obtain serum. Identified collection cards were left at room temperature for 4 h to complete drying and then sealed in plastic bags to be stored at room temperature for 3 months, and then at 4 °C for another 3 months before testing.

### Serological examination

The elution protocol and MAT serological technique for filter paper were adapted from Mercier et al. [44]. At first, for dry blood spot elution, two spots (5 mm diameter each) were punched from *Whatman™903* collection cards and placed in flat bottom microplates. Then, to yield a 1:8 dilution of serum, they were agitated overnight in 80 μl phosphate buffer saline pH 7.2 (BioMérieux, Marcy l’Etoile, France) at 400× *rpm* at room temperature. Sera from ordinary collection tubes and those obtained from eluted dried spots were tested for IgG antibodies against *T. gondii* by MAT. Samples were screened at four serial dilutions (1:20, 1:40, 1:100 and 1:800) with a cut-off dilution at 1:20.

The current study applied the MAT protocol of Desmonts & Remington [48]. We ensured that the final concentration of 2-mercaptoethanol for each dilution was 0.05 M after antigen addition. Serological controls for the filter paper adapted MAT were fresh blood from seronegative and experimentally infected seropositive Swiss mice (*Mus musculus*, Janvier, Le Genest-Saint-Isle, France) [44]. Although these control sera were not cat sera, they represent a quality control for each series of serological tests. Mouse blood with *T. gondii* antibodies were spotted onto a 5 mm diameter circle of filter paper, allowed to dry and stored in the same conditions as the samples.

### Statistical analysis

Statistical analyses were performed using logistic regression and Fisher’s exact test. Results were considered significant when *P* < 0.05. Statistical analyses were performed using the R × 64.3.3.0 software [49] with the two-tailed significance level of 5%.

## Results

Samples stored on filter paper showed the same qualitative results for MAT as sera from collection tubes. Positive samples (36) showed high titers (≥1:800) with both techniques, except for 3 cases which showed discordance between titers obtained with MAT on serum or dried blood spots (800/20, 100/40 and 100/800, respectively). Antibodies to *T. gondii* were detected in 4/107 (3.74%) stray cats from the private residential district (RD) and in 32/265 (12.08%) animals from the municipal shelter (MS).

The prevalence difference between the two localities was significant with both statistical tests (*P <* 0.01 with CI of 95%). Logistic regression quantified the difference in infection risk between the two localities. It showed that the risk of infection for cats was 3.54 times higher in MS compared to RD with a 95% confidence interval from 1.22 to 10.26. (*P* < 0.05). No statistically significant association regarding the age or sex of animals was observed (Table 3). Regarding the reproductive status, the association with prevalence was not statistically significant if we consider the whole population of cats, but became significant if we consider only RD cat population (*P* = 0.048).

**Table 3:** MAT results in the different cat populations and risk factors

Location	Entire population	Gender^a^		Reproductive status			Age class	
		Female	Male	Neutered	Not neutered	NI	Adult	Juvenile
MS	32/265 (12.08) [8.40–16.60]	20/163 (12.27) [7.65–18.30]	12/101 (11.88) [6.20–19.80]	23/196 (11.73) [7.58–17.10]	1/4 (25) [0.63–80.60]	8/65 (12.31) [5.40–22.80]	32/257 (12.45) [8.67–17.10]	0/8 (0) [0.00,36.90]
RD	4/107 (3.74) [1.02–9.30]	3/68 (4.41) [0.92–12.40]	1/39 (2.56) [0.06–13.50]	4/42 (9.52) [2.65–22.60]	0/43 (0) [0.00–8.20]	0/22 (0) [0.00–15.40]	3/91 (3.30) [0.69–9.30]	1/16 (6.25) [0.16–30.20]
*P*-value	< 0.01	ns	ns	ns	ns	ns	< 0.01	ns

*MS* Municipal shelter, *RD* Residential district, *NI* the reproductive status was not identified in clinical examination, *ns* not significant

^a^Sex could not be determined for one cat

Results are expressed as number of positive cats/total number of cats (% positive) [95% confidence interval]

## Discussion

This is the first time that MAT results from cat blood samples stored on filter papers were compared to those from sera samples. In fact, it was expected that MAT would have been less sensitive when performed on filter paper samples due to loss of detectable antibodies during storage and elution. Nevertheless, the differences observed for three sera did not change the prevalence results and may be due to the high antibody levels and short duration of sample storage [39, 44]. It certifies the accuracy of dried blood samples of cats tested by MAT and confirms this technique as a reliable alternative storage method.

Moreover, filter papers proved to be appropriate for the unfavorable conditions of sample collection from feral cats in which restraint requires speed by the health staff. Taking this into account, the possibility of testing with only a few drops of blood without sedation or animal stress provides us the necessary speed to ensure safe and effective sample collection. Moreover, it corroborates advantages highlighted by other studies, such as the impossibility of frozen or refrigerated storage of sera [40, 42]. It is also convenient if sample transport is subject to unfavorable conditions, high cost or restrictions, as in remote and isolated areas [38, 39]. It should be noted that our samples were stored up to 6 months, first at room temperature, and then at 4 °C, without silica gel. Nogami et al. [50] already noted a successful long-term preservation (up to 12 months) of feline anti-*T. gondii* antibody activity on filter paper strips stored either at 25 °C or 4 °C, but preservation was improved when filter papers were stored with silica gel.

Others studies concerning *T. gondii* infection in cats in the region of Rio de Janeiro used a different serological test making it difficult to compare results [51–54]. However, the prevalence of *T. gondii* antibodies in cats from this study seemed to be lower than those found in other populations in Brazil with the use of MAT (Table 1). Despite the use of the same serological test in all these studies, a remarkable range of results was observed. These different results are probably due to sample collection from markedly different environments.

There are possible biases to explain the significantly higher seroprevalence in MS compared to RD. First, given the difficulties of trapping animals, sampling of 50% of RD estimated population probably consisted of more stray cats than feral ones. Thus, the data not included in the study may have caused an underestimation of prevalence due to different feeding habits. The wild cats have a diet based mostly on hunting, while more sociable cats are used to being fed with kibbles occasionally provided by residents. Supplement feeding in urban stray cat populations may reduce cat exposure to toxoplasmosis [36]. Secondly, cats of RD were not neutered systematically, as in MS population, so an excessive number of kittens (15%) took part in sampling at this location. As the opportunity to acquire *Toxoplasma* infection through predation is lower for kittens, this could have explained the lower prevalence in RD cats. Nevertheless, no statistically significant association regarding the age of animals was observed. When kitten samples were removed for new statistical analysis, there remained a significant difference between the two areas (*P* = 0.006) (data not shown).

Usual risk factors for *Toxoplasma* infection associated with cat populations are stray *vs* owned cats, sex, or sterilization as these factors may have an influence on predation and social behavior [2]. These risk factors were not significant in our study. Sheltered cats may be considered as owned animals (restricted area, manufactured food and human interaction). Results from this study diverged from those of other teams that suggested that stray cats generally have a higher prevalence than owned ones [33, 55–59]. No significant association was observed regarding sex and *T. gondii* infection in cats from both populations. This result differs from those from Afonso et al. [60] which suggested that males are more infected than females when more prey is caught due to male predation efficiency. Once more, these findings lead us to suspect that the non-exhaustive sampling at RD could have biased data since males generally are more feral and difficult to trap compared to females. Since virtually all cats were neutered at MS, the association of reproductive status and risk factor was verified based only on the RD population. Similar to results reported by Afonso et al. [56], there was a positive correlation between *T. gondii* infection and sterilization within the RD cat population. It would be prudent to acquire additional samples to confirm this association. Neutered cats are more social and less wandering. This characteristic may have a protective effect since roaming may increase risk of infection by predation [36]. However, the increased risk of infection by oocysts may also be considered since social groups share restricted areas and common defecation sites [61, 62].

The positive association of *T. gondii* infection and the location where cats live corroborates the hypothesis that antibody prevalence in cats may vary according to different environments [36, 59]. In locations surveyed in this study, the associated environmental risk factors could be cat density, diversity of prey, and climatic conditions. Concerning the density risk factor, it is quite discrepant: 31 m^2^/cat at MS and 3587 m^2^/cat at RD. Considering that a cat can excrete millions of oocysts during early infection [2], we can deduce that high densities of cats in the downtown area must have contributed to local environmental contamination favoring oocyst transmission to intermediate hosts or between cats [36, 63]. In contrast, low densities may also contribute to infection, if predation rate is favorable [36]. At RD, we observed a low density of cats and a high biodiversity featuring abundance of prey. Different prey species have different infection rates according to their susceptibility, exposure and lifespan [60]. Present at RD, Brazilian guinea pigs are possibly one of the major food source for cats and other local carnivores. Little is known about natural infection by *T. gondii* for this species. Pelecaniformes (herons, egrets and ibis) and Capybaras have significant prevalence of *T. gondii* antibodies in other Brazilian environments [29, 33, 64, 65]. But even if they are plentiful at RD, they are improbable prey for feral cats. Another hypothesis that should be considered is the significant presence of crustaceans and fish at RD mangroves which serve as an estuary. This abundant and easy food source for local feral cats may decrease the risk of infection as they are not intermediate hosts for *T. gondii* [2].

Climatic factors influence the risk of infection in cats and their prey [56, 63, 66, 67]. Oocyst survival is well known to depend on physical and climatic conditions [56, 68]. Despite the short distance between MS and RD, factors such as the proximity to the sea, mountain ranges and vegetation determine climatic variations between these two close areas. RD is in a humid subtropical region of Rio de Janeiro. In the winter, moist winds from the sea are buffeted by mountains, which increase the amount of rainfall. This region usually receives almost twice the volume of rain compared to the downtown district of MS [69]. In the current study, the rainfall index seems to have a negative association with *T. gondii* infection, which differs from that observed by Afonso et al. [56]. In RD, local high precipitation and mangroves may favor runoff and vegetation uptake of oocysts [24, 70–73].

## Conclusion

In conclusion, this study suggests that differences in environmental features and human deterioration of habitats may play a role in cat infection levels probably through soil contamination by *T. gondii* and cat feeding behavior. The high local densities of cats in public shelters provide a high concentration of oocysts in areas close to humans. Further studies in tropical environments are necessary to elucidate this question. These studies may be facilitated by using dried blood spots on filter paper which was shown to be an advantageous approach for serological surveys of *T. gondii* infection in cats.

## Abbreviations

DVM: Doctor of veterinary medicine
MAT: Modified agglutination test
MS: Municipal animal shelter
RD: Private residential district
